# Summer and Autumnal Diseases for 1840

**Published:** 1840-11

**Authors:** 


### THE WESTERN JOURNAL.

Vol. II.—No. XI.

LOUISVILLE, NOVEMBER 2, 1840.

### SUMMER AND AUTUMN DISEASES OF 1840.

In one of our early numbers, we took occasion to call our readers attention to the fact, that the years 1838 and 39 had been uncommonly dry; but that 1840 up to the time (May) when we wrote had been rainy. As the summer and autumn, generally, have been wet, the year may be characterized as one of great humidity. In the article to which we have referred, it was suggested that a more favorable opportunity for estimating the comparative influence of dry and rainy seasons, would be furnished by these extremes, than was likely to occur again very soon.

We now ask, in what respects have the summer and autumnal diseases of the present year, differed from those of the two preceding? This question ought to be answered, and can be answered better at this time than hereafter, for memorandum books never become more copious, nor memories more accurate by time.

In our travels, for medical observation, in the State of Ohio, we have ascertained that the towns have been more healthy than the country; and, while the latter has not suffered any great *mortality*, intermittent and remittent fevers have had rather an extensive prevalence. Again we respectfully and earnestly solicit from our brethren a state of their observations on these and our other summer and fall endemics. D.

